# Integrating metagenomics and culturomics to uncover the soil bacterial community in *Asparagus cochinchinensis* cultivation

**DOI:** 10.3389/fmicb.2024.1467864

**Published:** 2024-12-04

**Authors:** Jingsheng Yu, Shuai Yang, Xiaoyong Zhang, Xiongwei Liu, Xuebo Tang, Liuyan Wang, Jinglan Chen, Huimin Luo, Changmin Liu, Chi Song

**Affiliations:** ^1^Institute of Herbgenomics, Chengdu University of Traditional Chinese Medicine, Chengdu, China; ^2^Traditional Chinese Medicine Health Industry Promotion Center of Dongxing District, Neijiang, China; ^3^Neijiang Dongxing District Bureau of Health, Neijiang, China; ^4^Committee of Education, Science, Culture and Health of Dongxing District, Neijiang, China

**Keywords:** medicinal plant, *Asparagus cochinchinensis*, soil bacterial community, metagenomics, culturomics

## Abstract

*Asparagus cochinchinensis* is a medicinal plant in China, which has gained attention owing its protective effect in human health. However, there are seldom studies to systematically reveal the rhizosphere bacterial community of *A. cochinchinensis*. In this study, we employed metagenomics and culturomics to analyze the bacterial community composition and diversity in continuous rhizosphere soil of *A. cochinchinensis*. Meanwhile, we assessed the effect of soil physicochemical properties on the bacterial community. Results showed that the most abundant TAXA is a taxon belonging to the family Streptomycetaceae, the genus *Mycobacterium* and the species *Oligotropha carboxidovorans*. The bacterial communities across various areas were similar. Significant differences of exchangeable magnesium and available phosphorus level were observed between three groups. Furthermore, bacterial community structure correlated closely with soil physicochemical properties. Additionally, a total of 103 strains were isolated and identified, representing 28 species. Based on this study, the rhizosphere bacterial community of *A. cochinchinensis* might influence its growth and development. The rhizosphere strains were isolated and their function request further investigation. This study firstly revealed the bacterial community in the *A. cochinchinensis* rhizosphere soil, providing valuable references for its quality improvement in practical cultivation process.

## Introduction

*Asparagus cochinchinensis* is a renowned medicinal plant belonging to Liliaceae family. Its health benefits have elucidated wide attention in recent years, with application in treating coughs, promoting skin whiten, and anti-aging effects (Lee et al., 2017; Wang et al., 2022). Research by Zhang L. et al. (2021) highlighted the potential of exosome-like nanovesicles from *A. cochinchinensis* for antitumor therapy. Furthermore, *A. cochinchinensis* is utilized in the fermentation of wine (Kim M. et al., 2017; Kim N. G. et al., 2017). Therefore, the market demand of *A. cochinchinensis* shows an increasing trend in recent years. *A. cochinchinensis* was mainly distributed across east and south Asia including China, Vietnam, Japan, and Korea. Now, China has been one of the main producing countries and exporters worldwide. The first record of *A. cochinchinensis* in China could date back to more than 2000 years ago in the Han Dynasty in Shennong’s Classic of Materia Medica (*Tianmendong* in Chinese). Notably, it is listed in the Pharmacopoeia of the People’s Republic of China (2020 edition, Chinese Pharmacopeia Commission, 2020, pp. 56–57). As a result of the suitable environment, Neijiang city (in Sichuan province with latitude 29°1 “to 30°2” north and longitude 104°16 “to 105°26” east) has become one of the main production cities of *A. cochinchinensis* in China. It is essential to develop efficient strategy to ensure the quality improvement of *A. cochinchinensis*.

Increasing number studies underscore the pivotal role of soil microorganisms in the growth and development of medicinal plants (Yu et al., 2024). For instance, Feng et al. (2021) studied the effect of *Bacillus* on the phthalides accumulation in *Angelica sinensis* (Oliv.), and observed that the *Bacillus* strains, which were isolated from rhizosphere soils, directly stimulated plant growth and the biosynthesis of butylidenephthalide. Wei et al. (2020) reported that microbial inoculants combined utilization of microbial inoculant and with garbage enzyme effectively reduced cadmium (Cd) uptake in *Salvia miltiorrhiza* by 37.90%, compared with the control group. Moreover, they might also facilitate the microbial remediation of soil contaminated with Cd. Csorba et al. (2022) investigated the interaction between *Alkanna tinctoria* and soil microorganisms and found positive correlations between alkannin levels and the relative abundances of *Labrys*, *Allorhizobium*, *Neorhizobium*, *Pararhizobium*, *Rhizobium*, and *Penicillium*. The advancement of metagenomics and culturomics has facilitated comprehensive exploration of the rhizosphere microbiota and its interaction with medicinal plants. Lian et al. (2023) simultaneously used metagenomics and culturomics technology to reveal the structure of rhizosphere microorganisms in Sinai desert farming systems and indicated that ecosystem functions of rhizosphere microorganisms from different sampling areas were similar although the microbial community structure and diversity were different. However, systematic studies on the interaction between the rhizosphere bacterial community and *A. cochinchinensis* are notably scarce.

In this study, we applied metagenomics and culturomics technology to comprehensively characterize the rhizosphere bacterial community of *A. cochinchinensis*. Concurrently, we analyzed the physicochemical properties of the rhizosphere soil from *A. cochinchinensis*. Our findings firstly revealed the rhizosphere bacterial community composition and isolated the rhizosphere bacterial strains from the *A. cochinchinensis* rhizosphere soil samples. Meanwhile, the effect of physicochemical properties on the rhizosphere bacterial community was evaluated, which provided references for the application of synthetic community for *A. cochinchinensis*.

## Materials and methods

### Sample collection

The soil samples were collected from the Neijiang City, Sichuan Province, China. Nine rhizosphere soil samples were collected from Yangjia Town (*n* = 3, YR), Shuangcai Town (*n* = 3, CR), and Guobei Town (*n* = 3, SR), and we also collected three non-rhizosphere soil samples from Yangjia Town (*n* = 3, YNR). The detailed collection information was listed in Table 1. The rhizosphere areas have a subtropical climate with an average annual temperature ranging from 15 to 28°C. The relative humidity annually averages around 80% and the annual rainfall is about 1,000 mm. The sampling procedure was as follows: approximately 2–3 cm of surface soil was initially removed to filter out plant detritus. Subsequently, rhizosphere soil located within 0.2 cm of the root was filtered through a 100-mesh screen and placed into sterilized bags. The samples were promptly submerged in liquid nitrogen immediately and stored at −80°C.

**Table 1:** Information for the *Asparagus cochinchinensis* rhizosphere soil samples in this study.

Sample number	Group	Sampling date	Sampling area	Altitude (meters)	Temperature (°C)	Relative humidity	SAMN number
CR1	CR	2023-6-12	China: Neijiang (29.749611 N 105.109195 E)	372.6 m	15–28	81%	SAMN41811701
CR2	CR	2023-6-12	China: Neijiang (29.749611 N 105.109195 E)	372.6 m	15–28	81%	SAMN41811702
CR3	CR	2023-6-12	China: Neijiang (29.749611 N 105.109195 E)	372.6 m	15–28	81%	SAMN41811703
SR1	SR	2023-6-12	China: Neijiang (29.525142 N 105.163067 E)	318.5 m	15–28	81%	SAMN41811704
SR2	SR	2023-6-12	China: Neijiang (29.525142 N 105.163067 E)	318.5 m	15–28	81%	SAMN41811705
SR3	SR	2023-6-12	China: Neijiang (29.525142 N 105.163067 E)	318.5 m	15–28	81%	SAMN41811706
YNR1	YNR	2023-6-12	China: Neijiang (29.766105 N 105.353238 E)	393.3 m	15–28	81%	SAMN41811707
YNR2	YNR	2023-6-12	China: Neijiang (29.766105 N 105.353238 E)	393.3 m	15–28	81%	SAMN41811708
YNR3	YNR	2023-6-12	China: Neijiang (29.766105 N 105.353238 E)	393.3 m	15–28	81%	SAMN41811709
YR1	YR	2023-6-12	China: Neijiang (29.766105 N 105.353238 E)	393.3 m	15–28	81%	SAMN41811710
YR2	YR	2023-6-12	China: Neijiang (29.766105 N 105.353238 E)	393.3 m	15–28	81%	SAMN41811711
YR3	YR	2023-6-12	China: Neijiang (29.766105 N, 105.353238 E)	393.3 m	15–28	81%	SAMN41811712

### Determination of the physicochemical properties of the *Asparagus cochinchinensis* rhizosphere soil

The analysis of pH, total nitrogen, total phosphorus, total potassium, organic carbon, available phosphorus, available potassium, exchangeable calcium, and exchangeable magnesium in the continuous rhizosphere soil samples was conducted as follows. Approximately 10 g of dried soil samples were placed into a 50 mL tube with 25 mL of distilled water and mixed for 1 min. After allowing it to settle for 30 min, pH was measured using a pH meter (PB-10, Sartorius, German). Approximately, 0.6 g of dried soil samples were analyzed using a Seal Auto Analyzer 3 Continuous Flow Analyzer (Germany) for total nitrogen, nitrate nitrogen, and ammonium nitrogen content. Total phosphorus and available phosphorus were quantified using an ultraviolet spectrophotometer (UV-2450, Shimadzu, Japan), while total potassium and available potassium levels were determined using a flame photometer (Shanghai, China). Exchangeable calcium and magnesium content were measured using an atomic absorption spectrophotometer (Hitachi Z-2000, Japan). The experimental procedures followed the methods outlined by Lu (2000).

### DNA extraction, metagenomic sequencing and bioinformatic analysis

Approximately 1.0 g of frozen soil sample was transferred into a sterilized 15 mL centrifuge tube containing 1.5 g of glass grinding beads. Bacteria DNA products were extracted as the instruction of the EZNA^®^ Soil DNA Kit (D5625, Omega Bio-Tek., Inc.) manufacturer. Purity and concentration of the extracted DNA were assessed using 2% agarose gel electrophoresis and Nanodrop (ND ONE, Genes Ltd.). A library was prepared using the NEB Next^®^ UltraTM DNA Library Prep Kit for Illumina (NEB, United States). Qualified DNA samples were randomly fragmented into 350 bp fragments using a Covaris (Covaris S2 System, Massachusetts, United States) ultrasonic fragmentation instrument. A complete library was constructed as follows: terminus repair, polyA tailing, sequence linking, purification, and PCR amplification. Finally, the AMPure XP system was used to purify the PCR products, and an Agilent 2100 was used to determine the insert size of the library. The real-time PCR was used for quantitative analysis of the library concentration. The indexed coding samples were clustered on the cBot Cluster Generation System using the Illumina PE Cluster Kit (Illumina, United States) according to the manufacturer’s instructions. Purified DNA samples were stored at −20°C. Subsequently, DNA sequencing was performed on the Illumina NovaSeq 6000 platform. The raw data was deposited to the National Center for Biotechnology Information Sequence Read Archive database with accession numbers SAMN41811701- SAMN41811712.

The quality control and bioinformatics analysis were conducted as follows. The low-quality data was filtered using fastp and Samtools software. Clean sequences were assembled into contigs using MEGAHIT software (v. 1.2.9, −k-list 21, 29, 39, 59, 79, 99, 119, 141 –min-contig-len 500, v1.2.9, Li et al., 2015). The Prodigal package within Prokka (v. 1.14.6) software were employed to predict ORF genes (Seemann, 2014). Predicted proteins encoded by these genes were functionally annotated by blasting against the UniProt, KEGG, and GO database to infer their biological functions (Camacho et al., 2009; Finn et al., 2011). Clean reads were directly determined with Kraken2 (v. 2.1.2) gene sets to identify bacterial species (Wood et al., 2019). To compare the gene abundance differences among different groups, the number of genes were originally standardized. The TPM (Transcripts Per Million) algorithm is used to standardize gene abundance. The DEseq2 was used to analyze the differentially expressed genes between various groups. The criteria for between various groups DEGs were |log2 (FoldChange)| > 1.5 and FDR < 0.01. Relative abundances of these species were calculated using Bracken (Lu et al., 2017). Alpha diversity indices (Chao 1, Shannon, Simpson, and richness) were computed to evaluate bacterial community diversity and richness using USEARCH (v. 10.0.240, Edgar, 2010, 2013). Rarefaction curves were calculated using USEARCH (v. 10.0.240) and bacterial community composition diagrams were generated using R software v.3.3.1 (Jami et al., 2013). The beta diversity was analyzed using the prcomp function in R software (v.3.3.1, Jami et al., 2013) and visualized using the ggplot2 package. To explore the interaction between soil bacterial communities and physicochemical properties, we conducted mantel test (R software) to perform association analysis.

### High-throughput isolation and identification of bacteria from the *Asparagus cochinchinensis* rhizosphere soil

Fresh soil samples from *A. cochinchinensis* were utilized to isolate and cultivate bacterial strains. Based on the previous reports by Zhang et al. (2018), we used multiple mediums (Tryptic soy broth medium, 02-34, Aobox Biotechnology, Beijing, China; Luria-Bertani medium; Nutrient Broth medium) for bacteria isolation and cultivation. The soil sample was transferred into a sterilized centrifuge tube with 9 mL of DEPC-treated water (cat. BL510B, LABGIC, Beijing, China) and subjected to oscillation cultivation at room temperature for 30 min with 30 rpm/min. Approximately 200 μL of culture supernatants were then diluted with 25 mL of 10 mM magnesium chloride solution and left at room temperature for 15 min to release bacteria. The diluted solution was cultivated into 96-well cell culture plates, with the concentration of diluted solution selected to ensure approximately 30% of the wells exhibited bacterial growth. Cultivation plates were subsequently incubated at room temperature for 14 days. After incubation, DNA products from bacterial strains were extracted using the TIANamp Bacteria DNA Kit (DP302, TIANGEN BIOTECH Co., Ltd., Beijing). Bacterial strains were identified by amplifying the 16S rRNA gene using designed primers 27F (5′-AGAGTTTGATCCTGGCTCAG-3′) and 1492R (5′-TACGGCTACCTTGTTACGACTT-3′, White et al., 1990). Identified high-quality strains were stored in 40% (vol/vol) glycerol at −80°C after three rounds of continuous streaking.

## Results

### Physicochemical properties of the *Asparagus cochinchinensis* rhizosphere soil

**Figure 1:**
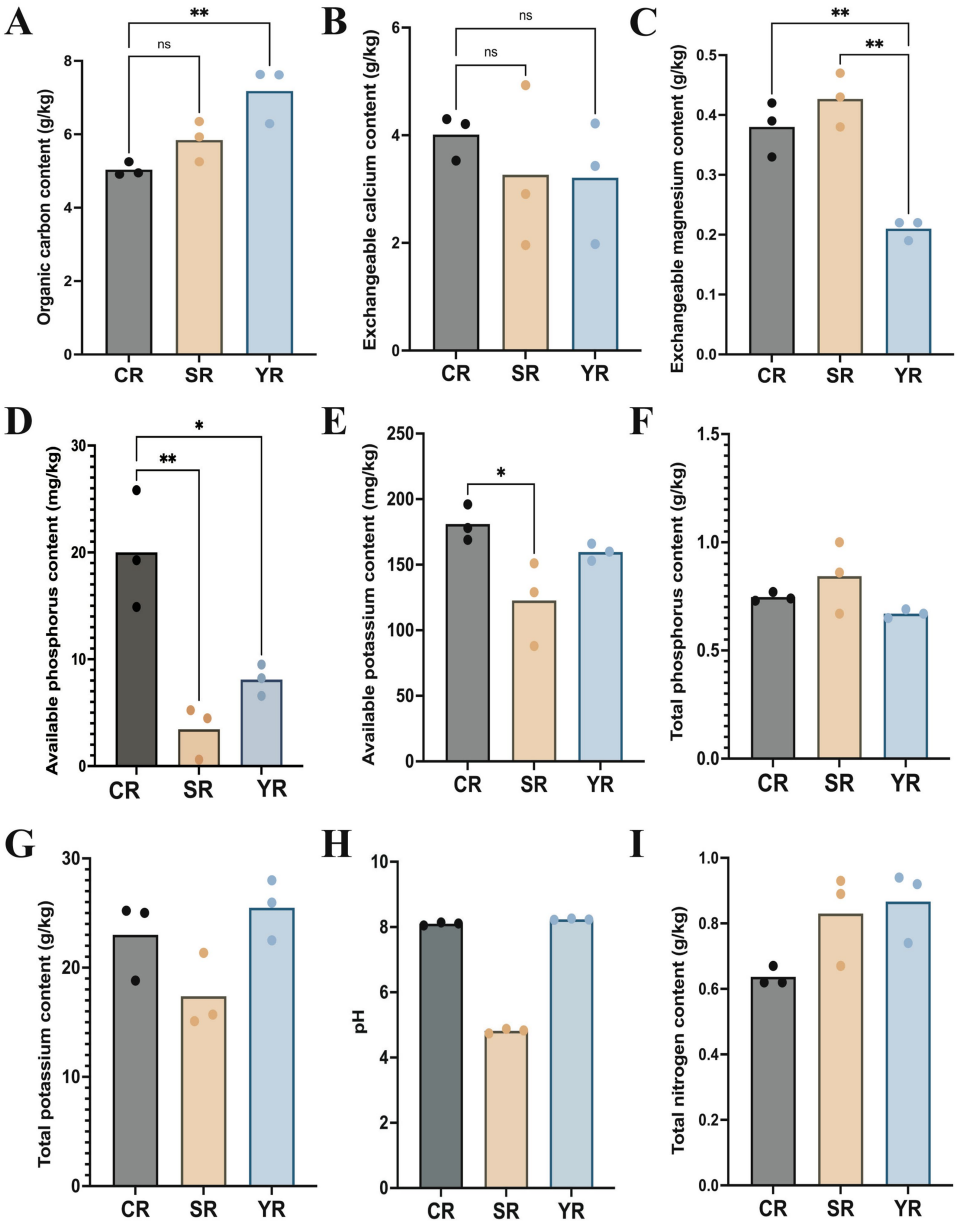
Physicochemical properties of the *A. cochinchinensis* rhizosphere soil. **(A)** Average organic carbon contents in the YR, SR, and CR group. **(B)** Average exchangeable calcium contents in the YR, SR, and CR group. **(C)** Average exchangeable magnesium contents in the YR, SR, and CR group. **(D)** Average available phosphorus contents in the YR, SR, and CR group. **(E)** Average available potassium contents in the YR, SR, and CR group. **(F)** Total phosphorus contents in the YR, SR, and CR group. **(G)** Total potassium contents in the YR, SR, and CR group. **(H)** pH in the YR, SR, and CR group. **(I)** Total nitrogen contents in the YR, SR, and CR group. * Represents *p* < 0.05, ** Represents *p* < 0.01.

The results showed that the average organic carbon content in the YR group (7.18 g/kg) was higher than that in the SR (5.84 g/kg) and CR group (5.03 g/kg, Figure 1A). Significant difference in carbon content was observed between the YR and CR groups (*p* = 0.0093). The exchangeable calcium content was higher in the CR group (4.01 g/kg) than that in the SR (3.27 g/kg) and CR group (3.21 g/kg, Figure 1B). The exchangeable magnesium level in the YR group (0.21 g/kg) was significantly lower than that in the SR group (0.43 g/kg, *p* = 0.0049) and CR group (0.38 g/kg, *p* = 0.0014, Figure 1C). The CR group (20.00 mg/kg) had higher available phosphorus contents than the SR group (3.44 mg/kg, *p* = 0.0034) and CR group (181.00 mg/kg, Figure 1D). The available potassium content in the CR group was higher than that in the SR (122.67 mg/kg) and YR group (159.67 mg/kg, Figure 1E). The total phosphorus content was highest in the SR group (0.84 g/kg), followed by the CR group (0.75 g/kg) and YR group (0.67 g/kg, Figure 1F). The total potassium result showed that the YR group (25.49 g/kg) had the highest level, followed by the CR group (23.02 g/kg) and SR group (17.39 g/kg, Figure 1G). The pH results indicated that all CR and YR samples in this study were alkaline, and SR samples were acidic (Figure 1H). The total nitrogen content in the YR group (0.87 g/kg) was higher than that in the SR group (0.83 g/kg) and CR group (0.64 g/kg, Figure 1I).

### Rhizosphere bacterial community in *Asparagus cochinchinensis* rhizosphere soil through metagenomics technology

#### Bacterial community composition in *Asparagus cochinchinensis* rhizosphere soil

**Figure 2:**
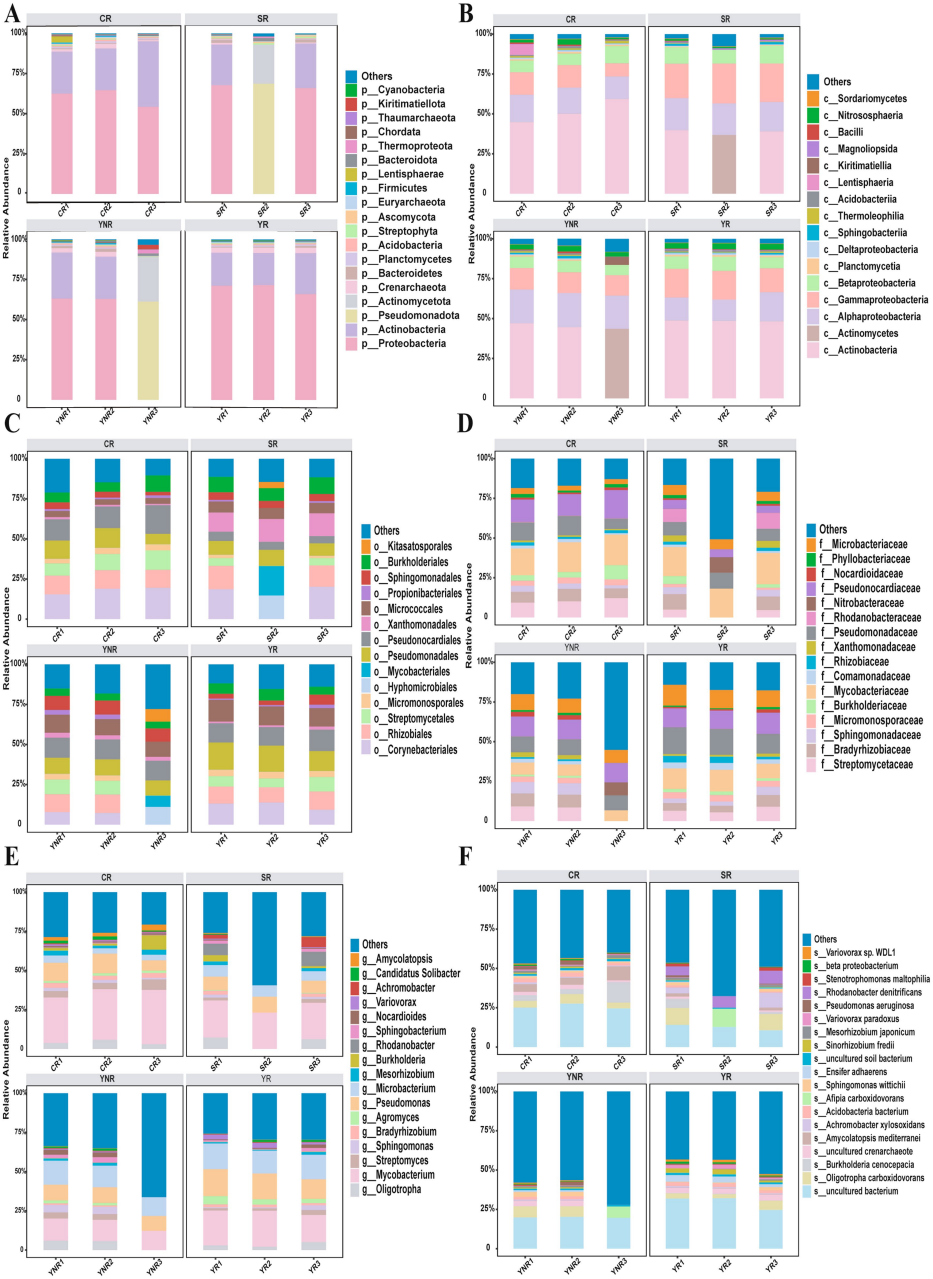
Bacterial community composition in the *A. cochinchinensis* rhizosphere soil at the **(A)** Phylum level. **(B)** Class level. **(C)** Order level. **(D)** Family level. **(E)** Genus level. **(F)** Species level.

The bacterial community in 12 *A. cochinchinensis* rhizosphere soil samples was sequenced using high-throughput sequencing technology. A total of 586,290,474 clean reads were obtained from 12 rhizosphere soil samples. Each sample yielded 103,629 assembled contigs. The raw sequences were deposited in the National Center for Biotechnology Information Sequence Read Archive database with accession numbers. Gene assemble results indicated that the contig numbers in each sample ranged from 65,454 to 221,127, all with GC content exceeding 60%. Alpha diversity index (Shannon, Chao1, Richness, and Simpson index) statistical analysis showed that significant differences of Shannon and Simpson indices were observed between various groups based on Anova (Table 2). Taxonomic annotation results showed that a total of 75 phyla, 166 classes, 410 orders, 706 families, 1,565 genera, and 4,424 species were identified in this study. At the phylum level, Proteobacteria, Actinobacteria, and Pseudomonadota were the most abundant with the relative abundances of 0–71.5%, 0–40.8%, and 0–68.7% (Figure 2A). Actinobacteria (0–59.3%), Corynebacteriales (0–19.5%), Actinomycetes (0–43.6%), and Streptomycetaceae (0–12.2%) were dominant at the class, order, and family level, respectively (Figures 2B–D). *Mycobacterium* (0–34.6%) was the most abundant at the genus level (Figure 2E). At the species level, *Oligotropha carboxidovorans* (0–34.6%), *Burkholderia cenocepacia* (0–2.2%), *Amycolatopsis mediterranei* (0–8.8%), *Achromobacter xylosoxidans* (0–3.8%), *Afipia carboxidovorans* (0–11.6%), *Sphingomonas wittichii* (0–3.1%), *Ensifer adhaerens* (0–0.9%), *Sinorhizobium fredii* (0–2.8%), *Mesorhizobium japonicum* (0–2.0%), Var*iovorax paradoxus* (0–0.2%), *Variovorax paradoxus* (0–0.2%), *Pseudomonas aeruginosa* (0–1.1%), *Rhodanobacter denitrificans* (0–0.3%), and *Stenotrophomonas maltophilia* (0–3.6%) were dominant among all species (Supplementary Table S1, Figure 2F). These findings provided a comprehensive overview of the bacterial composition in *A. cochinchinensis* rhizosphere soils.

**Table 2:** Alpha diversity indices in each sample in this study.

Sample number	Shannon	Chao1	Richness	Simpson
CR1	4.55	1,259	1,259	0.072
CR2	4.35	1,148	1,148	0.087
CR3	4.01	1,068	1,068	0.090
SR1	4.78	1,929	1,929	0.043
SR2	4.61	1,444	1,444	0.044
SR3	4.78	1,727	1,727	0.041
YNR1	4.96	1,433	1,433	0.050
YNR2	4.95	1,426	1,426	0.051
YNR3	5.14	2,100	2,100	0.048
YR1	4.04	733	733	0.117
YR2	4.07	739	739	0.111
YR3	4.81	1,648	1,648	0.069

### Comparison of bacterial community in different groups based on *Asparagus cochinchinensis* rhizosphere soil

**Figure 3:**
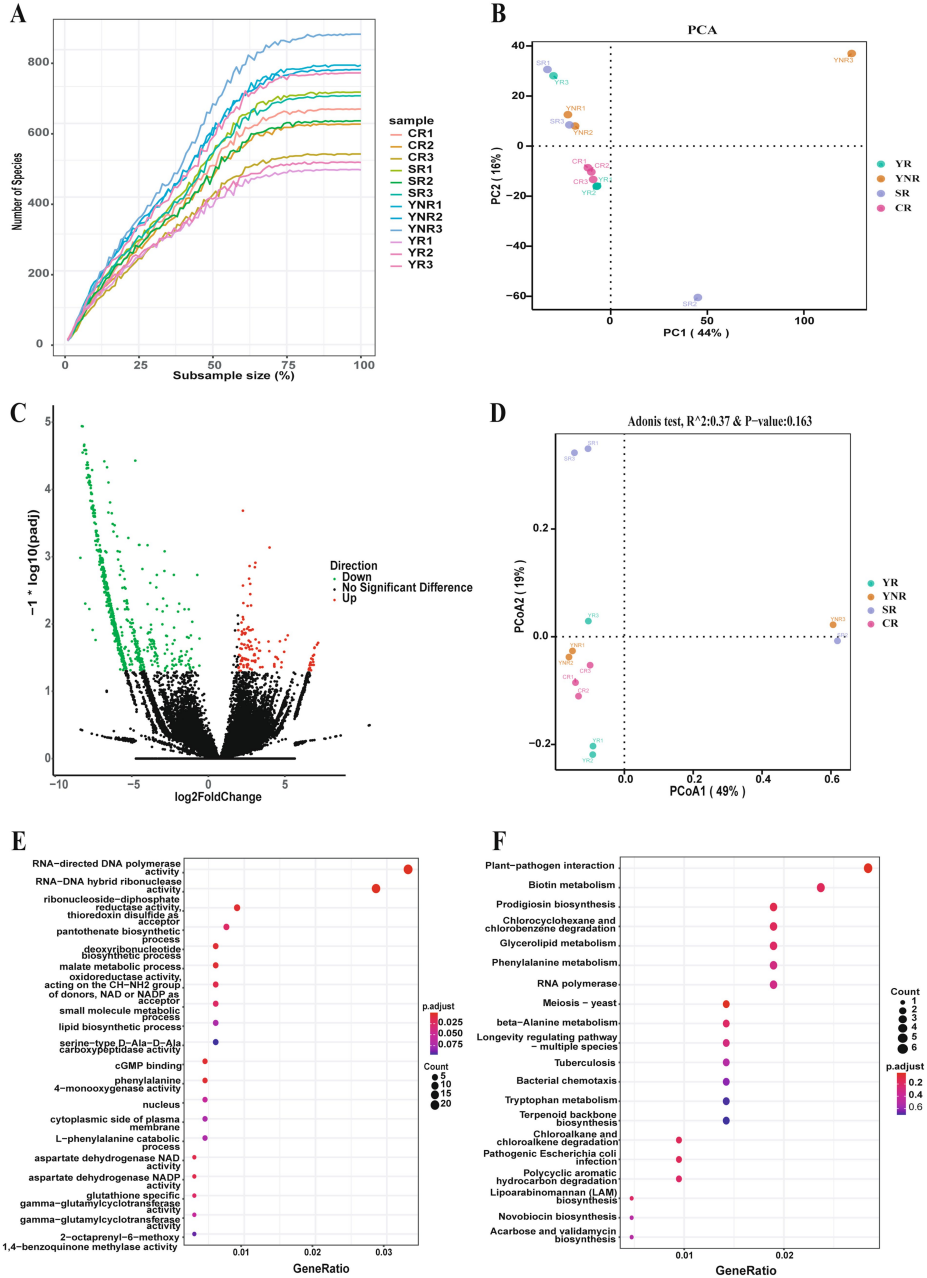
Comparison of the differences of bacterial community between various rhizosphere areas. **(A)** Rarefaction curve reveals the sequencing depth in each sample. **(B)** Principal Component Analysis of soil bacterial community among different groups. **(C)** Differentially expressed genes between various groups. **(D)** Principal co-ordinates analysis of soil bacterial community among different groups. **(E)** Gene function prediction of differentially expressed genes based on the Gene Ontology database. **(F)** Gene function prediction of differentially expressed genes based on the Kyoto Encyclopedia of Genes and Genomes database.

The rarefaction curve reflected that the sequencing data for each sample was sufficient to estimate the bacterial community (Figure 3A). Meanwhile, PCA and PCoA analysis further confirmed similarity of bacterial community composition across various areas (Figures 3B,D). Additionally, we compared differences of bacterial community between rhizosphere and non-rhizosphere soil groups. The average Chao 1 and Shannon indices in YNR group were higher than those in YR group (Table 2). The differences of bacterial composition were observed. At the genus level, the relative abundance of *Escherichia* in YR group was higher than those in YNR group. At the species level, YR group showed higher relative abundances of *Mycolicibacterium fortuitum*, *Lactobacillus paracasei*, *Agrobacterium rhizogenes*, *Rhizobium tropici*, *Pseudomonas lini*, *Acinetobacter johnsonii*, *Pseudomonas mosselii*, and *Pseudomonas umsongensis* (Supplementary Table S1). Gene expression difference analysis showed that there were 1,338 genes that expressed differently in YR and YNR group. One hundred and eighteen genes were up-regulated in YR group, and 1,220 genes were up-regulated in YNR group (Figure 3C). Differential gene enrichment analysis using KEGG Pathway showed that the genes of bacterial community function in YR group was mainly for plant-pathogen interaction, biotin metabolism, prodigiosin biosynthesis, meiosis-yeast, and pathogenic *Escherichia coli* infection. The function prediction result using Gene Ontology showed the bacterial community function in YR group was mainly for RNA–directed DNA polymerase activity, RNA–DNA hybrid ribonuclease activity, and cGMP binding (Figures 3E,F).

### Gene function annotation using various databases

**Figure 4:**
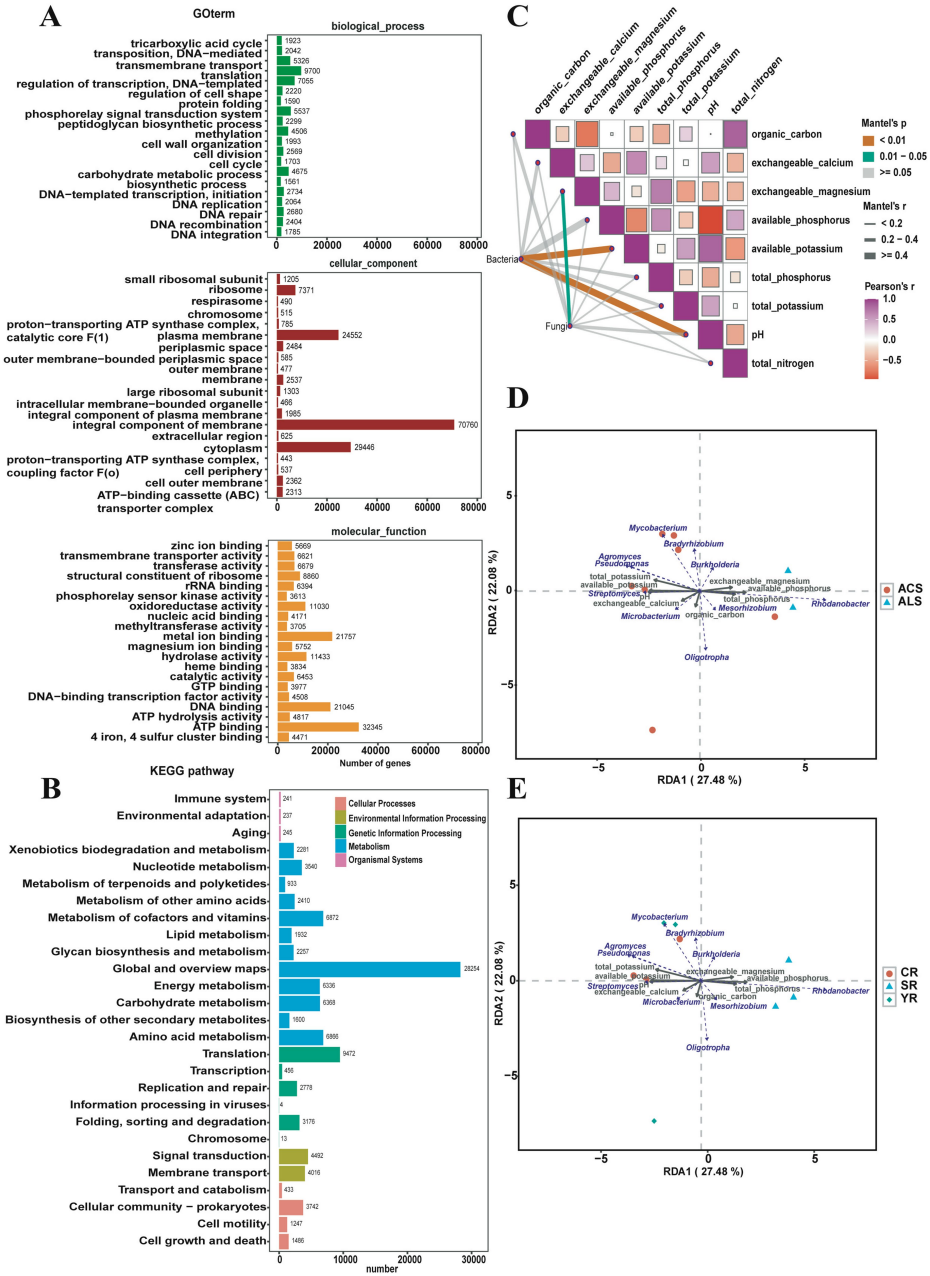
Function prediction of bacterial community and association analysis of physicochemical properties and soil bacterial community. **(A)** Function prediction of soil bacterial community based on the Gene Ontology database. **(B)** Function prediction of soil bacterial community based on the Kyoto Encyclopedia of Genes and Genomes database. **(C)** Association analysis between various physicochemical properties. **(D)** Mantel test for the analysis of relevance of pH and soil bacterial community. **(E)** Redundancy Analysis (RDA) of relationships between the relative abundance of top 10 bacterial genera and soil physicochemical factors in the YR, SR, and CR group.

The function of the bacterial community in *A. cochinchinensis* plantation soil was predicted using the KEGG and GO database. According to enrichment annotation, the bacterial community primarily contributes to global and overview maps, translation processes, and the metabolism of cofactors and vitamins. Gene enrichment annotation revealed that at the biological level, the bacterial community is involved in translation, phosphorelay signal transduction systems, and transmembrane transport. At the cellular level, integral components of membranes, cytoplasm, and plasma membrane played crucial roles. At the molecular level, ATP binding, metal ion binding, and DNA binding were the main function. Based on the COG database, the bacterial community function was predicted to be engaged in amino acid transport and metabolism, as well as energy production and conversion (Figures 4A,B).

### Association analysis between physicochemical properties factors and soil bacterial community composition

To explore the interaction between soil bacterial communities and physicochemical properties, we conducted mantel test (R software) to perform association analysis. The results revealed significant positive correlations (*p* < 0.01) between bacterial communities and pH, as well as available potassium activities. Additionally, Pearson’s correlation analysis indicated positive relationships among various physicochemical properties. Specifically, total nitrogen correlated positively with organic carbon and available phosphorus indices. pH levels showed positive associations with exchangeable calcium, available potassium, and total potassium indices. Furthermore, total phosphorus levels were positively correlated with exchangeable magnesium levels (Figure 4C). We employed Redundancy Analysis (RDA) to explore relationships between the relative abundance of top 10 bacterial genera and soil physicochemical factors. The results showed that the relative abundance of *Pseudomonas* exhibited a strong positive correlation with cumulative total potassium and available potassium. The relative abundance of *Streptomyces* was notably associated with cumulative pH levels, while the relative abundance of *Rhodanobacter* correlated with cumulative available phosphorus. Additionally, the relative abundance of *Burkholderi*a showed a significant relationship with cumulative exchangeable magnesium levels (Figures 4D,E).

### Isolation and identification of bacterial strains from the *Asparagus cochinchinensis* rhizosphere soil

**Figure 5:**
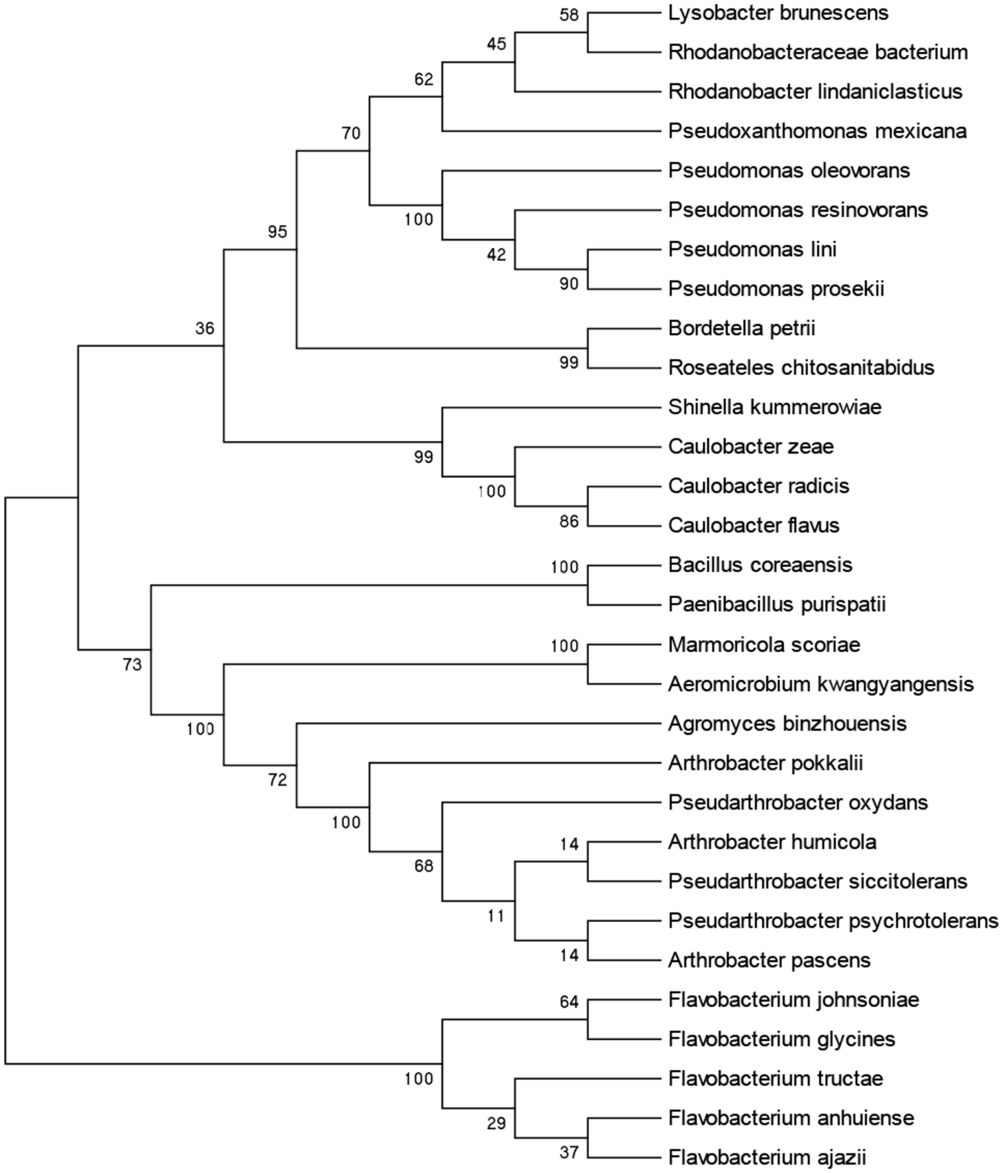
Phylogenetic tree based on 16S rRNA of bacterial strains isolated from *A. cochinchinensis* rhizosphere soil.

In this study, we used TSB, LB, and NB medium to isolate and identify the bacterial strains from the *A. cochinchinensis* rhizosphere soil. The strains were further identified through amplifying 16S rDNA region using the 27F/1492R primer. A total of 103 strains were isolated and identified, encompassing 30 species including *Agromyces binzhouensis*, *Arthrobacter pascens*, *Bordetella petrii*, *Aeromicrobium kwangyangensis*, *Rhodanobacter lindaniclasticus*, *Pseudarthrobacter oxydans*, *Marmoricola scoriae*, *Lysobacter brunescens*, *Arthrobacter pokkalii*, *Rhodanobacteraceae bacterium*, *Caulobacter zeae*, *Flavobacterium johnsoniae*, *Roseateles chitosanitabidusm*, *Caulobacter radicis*, *Pseudomonas lini*, *Flavobacterium ajazii*, *Pseudomonas resinovorans*, *Pseudoxanthomonas mexicana*, *Pseudomonas prosekii*, *Paenibacillus purispatii*, *Pseudomonas oleovorans*, *Flavobacterium anhuiense*, *Caulobacter flavus*, *Bacillus coreaensis*, *Shinella kummerowiae*, *Flavobacterium glycines*, *Flavobacterium tructae*, *Arthrobacter humicola*, *Pseudarthrobacter siccitolerans*, and *Pseudarthrobacter bacteria* (Figure 5; Supplementary Figure S1). All these strains were stored in institute of Herbgenomics, Chengdu University of Traditional Chinese Medicine for further experimentation.

## Discussion

Recent studies have extensively investigated the interaction between medicinal plants and soil bacterial communities. It has been reported that soil bacterial community might influence the metabolism of natural products in medicinal plants. However, the detailed mechanisms of these interactions require further exploration. Su et al. (2023) investigated the contribution of soil condition and microbiome in the accumulation of monoterpenes in the fruit of *Citrus reticulata* “Chachi” and they indicated that the rhizosphere microbiome enhanced terpene synthesis and increased the monoterpene level by affecting the host immune system. Zhang W. et al. (2024) conducted amplicon sequencing and metabolic profiling to analyze the relationship between soil microbiome and bisbenzylisoquinoline alkaloids in *Phellodendron amurense*. Results showed that there were seven bisbenzylisoquinoline alkaloids positively correlated with Nectriaceae and Sphingobacteriaceae. Similarly, Liu et al. (2022) reported direct regulation of glycyrrhizin and glycyrrhizic acid accumulation in licorice plants by the rhizosphere microbiome. Secondly, the soil microbiome might assist host medicinal plant in growth and resistance to biotic and abiotic stress. *Astragalus mongolicus* Bunge is a well-known medicinal plant and its cultivation requires amounts of nutrients. Shi et al. (2023) isolated and identified 86 strains form the root and rhizosphere soil samples of *A. mongolicus* and demonstrated that *Bacillus* sp. J1, *Arthrobacter* sp. J2, and *Bacillus* sp. G4 promoted the accumulation of nitrogen, phosphorus, and potassium content in *A. mongolicus*. Li et al. (2024) evaluated the effect of *Paenibacillus polymyxa* 7250 in combination with the symbiotic bacteria on ginseng. Results showed that this treatment improved ginseng yield and disease resistance through increasing *Rhodanobacter*, *Pseudolabrys*, and *Gemmatimonas*. Under low nitrogen conditions, Jiang et al. (2024) reported that the beneficial plant growth-promoting rhizobacteria improved the nitrogen utilization efficiency and regulated the synthesis of target furanocoumarins of *Angelica dahurica* var. *formosana*. In this study, we revealed the bacterial community of *A. cochinchinensis* rhizosphere soil through Illumina NovaSeq 6000 platform. Based on our results, *Mycobacterium*, *Pseudomonas*, *Microbacterium*, *Agromyces*, and *Bradyrhizobium* were the main genera in the soil samples. KEGG function prediction results showed that these genera were related to the immune system and environmental adaption. Several of these genera have been reported as beneficial microorganisms for plants (Cao et al., 2023; De Maria et al., 2011; Gao et al., 2024; Tsavkelova et al., 2024). Thus, to deepen our understanding of the interaction between these beneficial microorganisms, pathogens, and *A. cochinchinensis*, we isolated these strains from the *A. cochinchinensis* rhizosphere soil samples for further investigation. Based on the previous studies, 11 of these strains have been reported as functional rhizosphere bacterial strains. Benmrid et al. (2024) investigated the beneficial effect of multiple rhizobacteria on the wheat growth under drought conditions, and results showed that the incubation with *A. pascens* increased dry weight of shoot and root of wheat plants. Additionally, *A. pascens* had the ability to assisting host plants to resist soil salinity stress and enhance physicochemical properties (Guan et al., 2023; Ullah and Bano, 2015). *A. kwangyangensis*, *A. humicola*, *C. flavus*, *P. oxydans*, *F. anhuiense*, and *P. lini* were reported as biocontrol strains to promote the growth of multiple plants (Elshafie and Camele, 2022; Giannelli et al., 2024; Kim M. et al., 2017; Kim N. G. et al., 2017; Jeong et al., 2019; Muñoz Torres et al., 2021; Tian et al., 2024). Meanwhile, *A. pokkalii*, *P. lini*, *C. flavus* and *B. coreaensis* had the potential to resisting biotic and abiotic stresses (Anand et al., 2023; Barbaccia et al., 2022; Krishnan et al., 2016; Zhou et al., 2021).

In this study, we used a high-throughput isolation and identification method to enrich the bacterial strains from the *A. cochinchinensis* rhizosphere soil. This method was initially constructed based on the reports by Zhang J. et al. (2021) we further optimized and adjusted this method based on the characteristics of *A. cochinchinensis* rhizosphere soil. Previous applications of this method have successfully been adapted for diverse multiple conditions such as deep vadose zone and plant roots (Zhang L. et al., 2024; Zhang W. et al., 2024; Jin et al., 2022). However, the disadvantages of this method should be optimized in the further studies. On one hand, single medium was difficult to comprehensively isolate bacterial strains in the soil sample, therefore, we employed three media to isolate and identify strains in this work. We considered to use more media in further studies. On the other, we mainly focused on the aerobic bacteria, while the isolation and identification of anaerobic bacteria requires to be considered in the further study. In conclusion, continuous optimization of this method continuously to explore the mechanisms is crucial for advancing our understanding of soil microorganisms and their impact on the growth and development of medicinal plants.

The combined utilization of metagenomics and culturomics technologies revealed the rhizosphere bacterial community of *A. cochinchinensis*. In recent years, more and more reports have applied both technologies simultaneously to study the microbiome in various aspects including gut microbiome, environmental microbiome, and food microbiome (Lee et al., 2022; Li et al., 2023; Vacca et al., 2024). Compared with using metagenomics or culturomics technology alone, the combined application overcomes certain disadvantages. Firstly, the metagenomics sequencing data may provide references for the selection of mediums of culturomics. Almost all microorganisms (culture-dependent and culture-independent) might be identified through the metagenomics sequencing technology, researchers can select the relevant mediums to isolate and identify the specific strain for further studies. In this study, the selection of mediums was also based on the metagenomic results, which *Mycobacterium*, *Pseudomonas*, *Microbacterium*, *Agromyces*, and *Bradyrhizobium* were the main genera in this study. Moreover, the interaction network analysis and community function prediction result based on metagenome is an important indicator for the construction of synthetic microbial community in culturomics. Li et al. (2021) analyzed the rhizosphere and root bacterial community in *Astragalus mongholicus* and constructed a disease-resistant bacterial community based on the metagenome result through the culturomics platform. Results demonstrated that the synthetic bacterial community suppressed the root rot disease of *A. mongholicus* infected by *Fusarium oxysporum* and promoted the growth of *A. mongholicus*. Secondly, due to bias in metagenomic sequencing data, culturomics has the ability of correcting this bias. Culturomics can not only be used to isolate and identify microorganisms, but it also has the potential to discovering new taxa and reduce unassigned operational taxonomy units based on metagenomic analysis (Lagier et al., 2018). Additionally, the strains isolated through the culturomics platform can be studied through multiple detection methods (e.g., genomics, transcriptomics, proteomics, and metabolomics technology). Therefore, the combined utilization of metagenomics and culturomics technologies in microbiome studies has been widely recognized. The application of these two technologies provides both genome data and strain resources simultaneously. In future, the combined utilization of both technologies will provide more comprehensive and systematical insights for microbiome studies.

## Data Availability

The datasets presented in this study can be found in online repositories. The names of the repository/repositories and accession number(s) can be found in the article/Supplementary material.
