# Corrigendum: Integrating metagenomics and culturomics to uncover the soil bacterial community in *Asparagus cochinchinensis* cultivation

**DOI:** 10.3389/fmicb.2024.1545445

**Published:** 2025-01-07

**Authors:** Jingsheng Yu, Shuai Yang, Xiaoyong Zhang, Xiongwei Liu, Xuebo Tang, Liuyan Wang, Jinglan Chen, Huimin Luo, Changmin Liu, Chi Song

**Affiliations:** ^1^Institute of Herbgenomics, Chengdu University of Traditional Chinese Medicine, Chengdu, China; ^2^Traditional Chinese Medicine Health Industry Promotion Center of Dongxing District, Neijiang, China; ^3^Neijiang Dongxing District Bureau of Health, Neijiang, China; ^4^Committee of Education, Science, Culture and Health of Dongxing District, Neijiang, China

**Keywords:** medicinal plant, *Asparagus cochinchinensis*, soil bacterial community, metagenomics, culturomics

In the published article, there was an error in the **affiliations**. Affiliations 5 and 6 should not have been included.

The affiliations previously read as:

“Jingsheng Yu^1,2†^, Shuai Yang^1,3†^, Xiaoyong Zhang^4,5^, Xiongwei Liu^4,6^, Xuebo Tang^4^, Liuyan Wang^4^, Jinglan Chen^4^, Huimin Luo^4^, Changmin Liu^1^ and Chi Song^1*^

^1^Institute of Herbgenomics, Chengdu University of Traditional Chinese Medicine, Chengdu, China

^2^Traditional Chinese Medicine Health Industry Promotion Center of Dongxing District, Neijiang, China

^3^Neijiang Dongxing District Bureau of Health, Neijiang, China

^4^Committee of Education, Science, Culture and Health of Dongxing District, Neijiang, China

^5^Institute of Chinese Materia Medica, China Academy of Chinese Medical Sciences, Beijing, China

^6^School of Pharmacy, Guizhou University of Traditional Chinese Medicine, Guiyang, China”

The corrected affiliations read as:

Jingsheng Yu^1†^, Shuai Yang^1†^, Xiaoyong Zhang^2,3^, Xiongwei Liu^2,4^, Xuebo Tang^2^, Liuyan Wang^2^, Jinglan Chen^2^, Huimin Luo^2^, Changmin Liu^1^ and Chi Song^1^

^1^Institute of Herbgenomics, Chengdu University of Traditional Chinese Medicine, Chengdu, China

^2^Traditional Chinese Medicine Health Industry Promotion Center of Dongxing District, Neijiang, China

^3^Neijiang Dongxing District Bureau of Health, Neijiang, China

^4^Committee of Education, Science, Culture and Health of Dongxing District, Neijiang, China

The authors apologize for this error and state that this does not change the scientific conclusions of the article in any way. The original article has been updated.

