# Supplementary figures and images for: Integrating metagenomics and culturomics to uncover the soil bacterial community in Asparagus cochinchinensis cultivation
Source: Front Microbiol. 2024 Dec 4;15:1467864. doi: 10.3389/fmicb.2024.1467864 (PMC11652531; doi:10.3389/fmicb.2024.1467864)

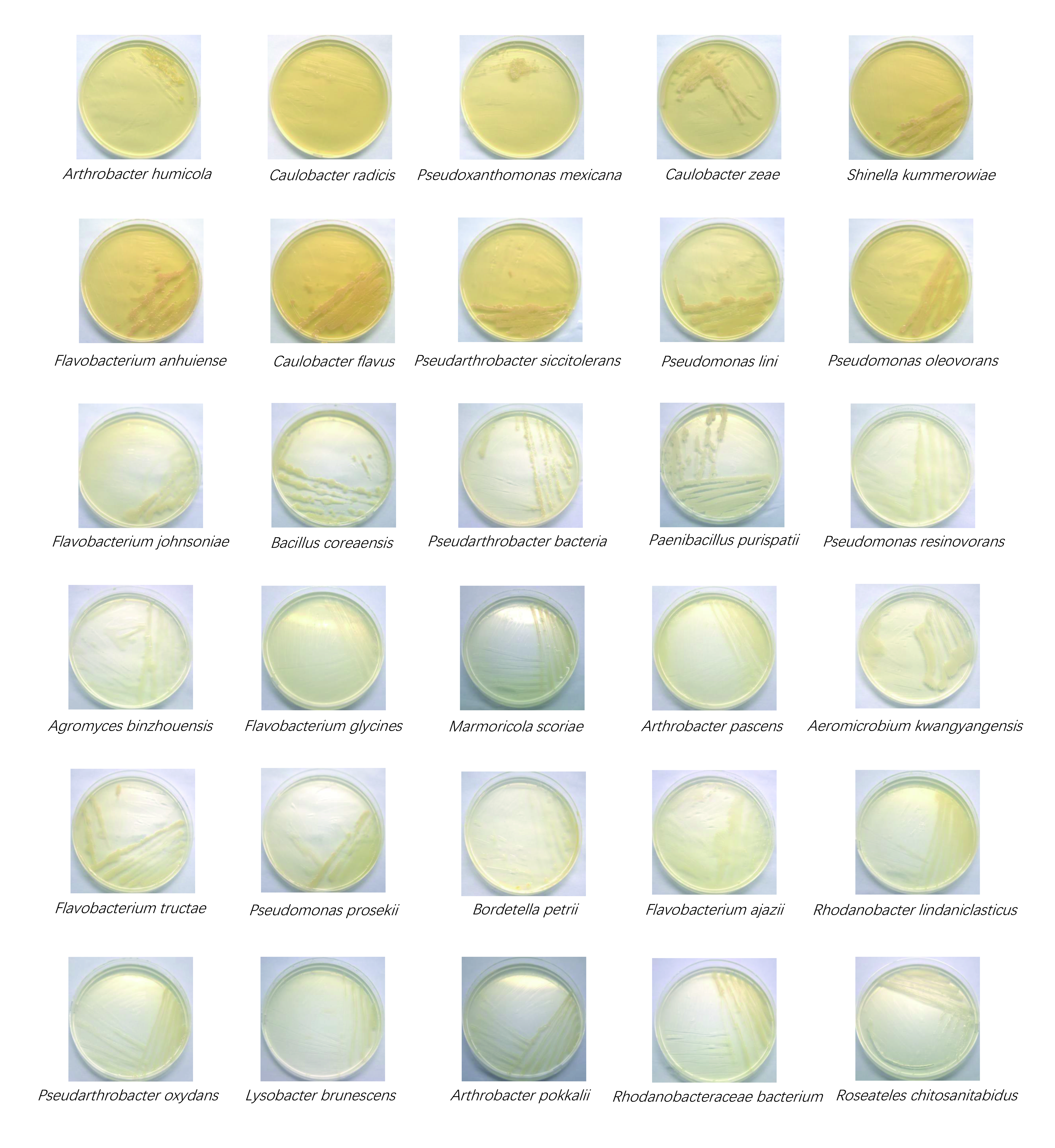

Supplement: Supplementary file 2 [file Image_1.TIFF]
